# Supplementary figures and images for: Identification of Candidate Genes for Root Traits Using Genotype–Phenotype Association Analysis of Near-Isogenic Lines in Hexaploid Wheat (Triticum aestivum L.)
Source: Int J Mol Sci. 2021 Mar 30;22(7):3579. doi: 10.3390/ijms22073579 (PMC8038026; doi:10.3390/ijms22073579)

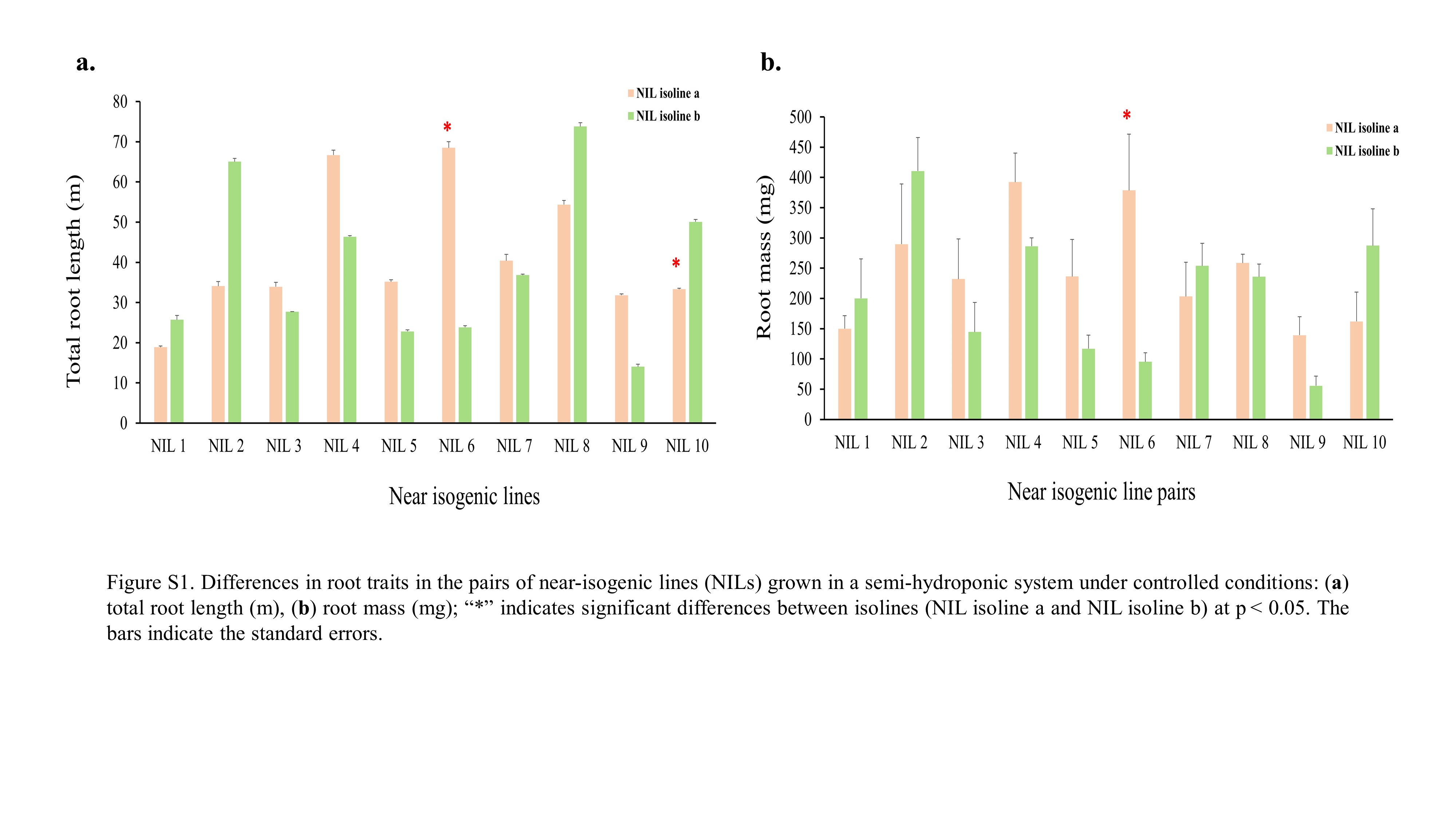

Supplement: Supplementary file 1 [file ijms-22-03579-s001.zip › ijms-1120734_Figure S1_Tanushree Halder_29 March 2021.jpg]

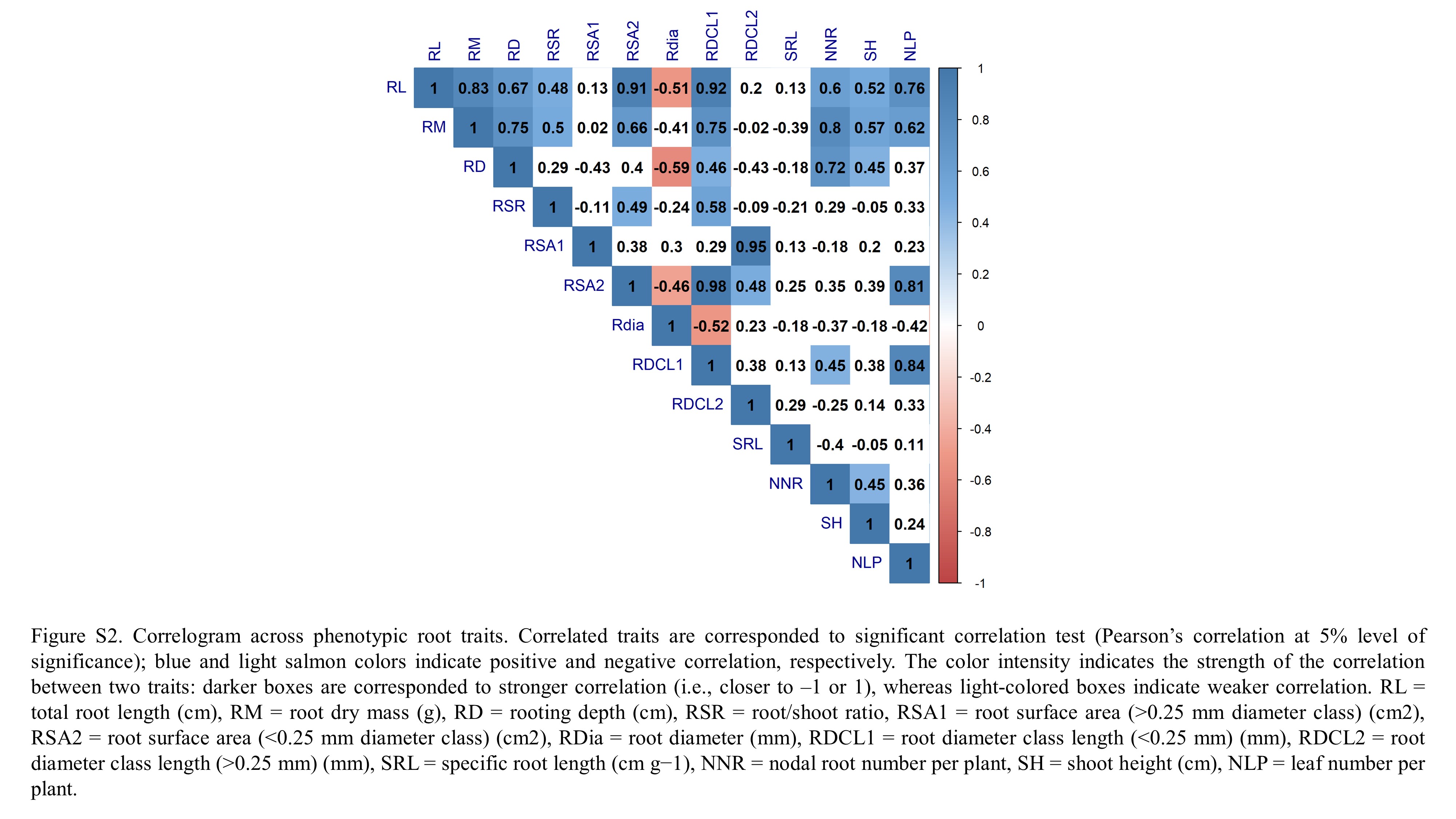

Supplement: Supplementary file 1 [file ijms-22-03579-s001.zip › ijms-1120734_Figure S2_Tanushree Halder_29 March 2021.jpg]
